# Pilot robotic mastectomy in Singapore (PRoMiSing I) study: first safety and feasibility prospective cohort trial in South East Asia

**DOI:** 10.1097/JS9.0000000000001674

**Published:** 2024-05-20

**Authors:** Chi W. Mok, Yert L. Melissa Seet, Zar C. Lin, Jun X. Jeffrey Hing, Chin M. Jaime Seah, Su-Ming Tan

**Affiliations:** a Department of Surgery, Division of Breast Surgery, Changi General Hospital; b SingHealth Duke-NUS Breast Centre, Singapore, Singapore

**Keywords:** breast cancer surgery, endoscopic breast surgery, minimally invasive breast surgery, pilot study, reconstruction, robotic mastectomy

## Abstract

**Background:**

Robotic mastectomy has been performed worldwide since 2015. The advantages of the robotic approach in nipple-sparing mastectomy have been proven with better visualization and preservation of blood supply to the nipple–areolar complex, with a lower incidence of necrosis. It also allows smaller incisions for both mastectomy and immediate breast reconstruction. To date, no centers in Singapore and Southeast Asia offer robotic mastectomy. We believe that robotic mastectomy is a feasible and safe technique that our population can utilize.

**Objectives:**

This study aimed to ascertain the surgical outcomes and perform a learning curve analysis in patients undergoing robotic mastectomy in a multi-ethnic South East Asian population.

**Methods:**

A single-arm prospective pilot study of eligible patients aged 21–70 years old with early breast cancer or high-risk patients indicated for risk-reducing mastectomy who were eligible and consented to robotic mastectomy were enrolled in this trial from December 22, 2022 to December 15, 2023.

**Results:**

A total of 29 consecutive robotic mastectomies were performed. The mean total operative time was 95±10.2 min. The average blood loss was 5.7±1.9 ml, and the average length of stay was 1.05 days. The mean mastectomy specimen weight was 251 g, and there was no conversion to conventional mastectomy in any case. Furthermore, there were no 30-day morbidity or complications in terms of wound infection requiring intervention, flap, and nipple–areolar complex necrosis, and postoperative hematoma/bleeding requiring intervention.

**Conclusion:**

This study contributes to the current evidence that robotic mastectomy is a safe and feasible option and could prove to be a great alternative to conventional mastectomy. Further prospective trials examining the long-term oncological outcomes of robotic mastectomy will be performed to establish the oncologic safety of this technique in breast cancer treatment.

## Introduction

Highlights

- Robotic mastectomy is a safe and feasible alternative to conventional mastectomy.
- In suitable patients with early breast cancer or those indicated for risk-reducing mastectomy, this study contributed to current evidence with no major complications, morbidities, or conversion.
- With appropriate training, the learning curve analysis showed a significant reduction in the total operative time and console time in the fifth and third cases, respectively.

Conventional nipple-and/or skin-sparing mastectomy (NSM/SSM) with or without immediate reconstruction is one of the standard surgical treatments^1^ for breast cancer or as a risk-reducing option for women at high risk of breast cancer. While this technique provides satisfactory oncologic and esthetic outcomes^1^, its disadvantages include skin flap and/or nipple–areolar complex (NAC) necrosis^1–3^, as well as a visible scar(s) on the breast.

In terms of technical aspects, NSM/SSM has inherent challenges in view of limited incisions and difficulties in dissection^4^. Since 2015, several institutions worldwide^5–9^ have adopted a new NSM/SSM technique using a robotic surgical system. Institutional experiences worldwide^10^ have demonstrated the feasibility and safety of this technique, coupled with improved patient satisfaction.

There are a few aspects of NSM and breast reconstruction that can be improved with the robotic approach. For example, better visualization and preservation of the blood supply to the NAC using a robotic surgical system would lead to a lower incidence of NAC necrosis, as shown in previous studies^11,12^. In addition, the robotic approach allows for smaller/fewer incisions for both mastectomy and immediate breast reconstruction. The demand for smaller/hidden incisions may be higher in Asian women, and infra-mammary fold incisions may not be suitable for women with smaller and nonptotic breasts, as shown in a meta-analysis looking at the health-related quality of life in breast cancer patients in Asia^13^.

To date, no center in Singapore offers robotic NSM/SSM (R-NSM/R-SSM). The investigators believe that robotic mastectomy is a feasible and safe technique that can be utilized in our population and provides superior esthetic outcomes with less morbidity and higher patient satisfaction.

This study aimed to conduct a single-arm prospective pilot study to investigate the surgical safety outcomes and learning curve analysis of robotic mastectomy in Singapore.

## Methodology

This prospective pilot study aimed to recruit at least 20 consecutive patients who underwent a robotic mastectomy. The study was registered on ClinicalTrials.gov. In addition, it has been reported in line with the STROCSS criteria^14^.

### Patient selection

Eligible patients aged 21–70 years old who fulfilled the inclusion and exclusion criteria were enrolled in this study. Written informed consent pertaining to the use of clinical records or perioperative pictures was obtained from each participant.

Preoperative breast ultrasound, mammography, and/or MRI were used to determine the eligibility of patients for robotic mastectomy. Computed tomography of the thorax, abdomen, and pelvis, as well as whole-body bone scan, may be used to exclude the possibility of distant metastases in the indicated cases.

### Inclusion and exclusion criteria

Inclusion criteria for robotic mastectomy included breast cancer (early-stage breast cancer, tumor size of <5 cm, no evidence of lymph node metastases, and no evidence of skin or chest wall invasion) and high-risk women indicated for risk-reducing mastectomy.

Exclusion criteria included patients with locally advanced breast cancer (with or without chest wall or skin invasion) or inflammatory breast cancer, extensive axillary lymph node metastasis (stage IIIB or later), high-risk patients with severe and poorly controlled comorbid conditions (including but not limited to diabetes, heart disease, renal failure, liver dysfunction), pregnant women, patients with previous thoracic radiation, and patients with any psychiatric, addictive, or any disorders that compromise the ability to give informed consent for participation in this study. In terms of a tumor to NAC distance, tumor with less than 5 mm distance to NAC, as shown in preoperative imaging, were excluded from the study.

The inclusion and exclusion criteria were based on current evidence derived from the existing literature on robotic mastectomy^15–19^.

### Conduct of robotic mastectomy

#### Preoperative marking and positioning

Preoperative marking was performed with the patient in standing and supine positions. After the induction of general anesthesia, the patient was placed in a supine position with the ipsilateral arm abducted to 90° to avoid conflict with the operative procedure.

#### Axillary staging procedure

In patients for whom sentinel lymph node biopsy (SLNB) is indicated, 3–5 ml of 1% methylene blue (Merck, Darmstadt, Germany) was injected into the breast parenchyma facing the ipsilateral axilla after the induction of general anesthesia. Breast tissue around the injection site was gently massaged for 5–10 min. SLNB was performed according to standard practices. Fresh frozen sections were sent for intra-operative analysis, as indicated. If the SLN was positive for metastases, complete axillary lymph node dissection up to level II was performed.

#### Docking and robotic mastectomy

A working space for the placement of a single port (Glove Port; Nelis Corporation, Gyeonggi-do, Korea) was created with a 3–4 cm subcutaneous flap dissected under direct vision. The tunneling technique was then used to facilitate skin flap dissection and create space between the skin flap and the breast parenchyma. Once adequate dissection was achieved, the single port was then inserted through the anterior axillary skin crease incision, and carbon dioxide (CO_2_) insufflation with air pressure maintained at 8 mmHg was used to create space for mastectomy. The ipsilateral shoulder was elevated to 30° to prevent conflict between the operating table and the docking of the robotic surgery system. The robotic side cart (da Vinci; Intuitive Surgical, Sunnyvale, CA, USA) was then positioned from the contralateral side or over the patient’s head, with the two robotic arm endoscopes extending over the patient in proximity to the ports before the ports were docked to the robotic arms. Subsequently, the operation was shifted to the da Vinci Xi (Intuitive Surgical) robotic platform controlled by the operating surgeon at the console. A 30° 12 mm diameter camera (Intuitive Surgical, Denzlingen, Germany) in the upper port was used to prevent collisions with other instruments. Dissection was performed using 8 mm monopolar scissors (Intuitive Surgical). Traction and counter-traction, along with maintaining exposure, were carried out using an 8 mm ProGrasp forceps (Intuitive Surgical). The location of the scissors and ProGrasp forceps can be changed intervariably during the operation. Dissection was first initiated from the superficial skin flaps by dissecting the septa between the skin flap and parenchyma created by the tunneling technique using monopolar scissors. A sub-areolar biopsy and fresh frozen section analysis was performed in NSM. If cancer cell invasion was found in the sub-areolar area, the entire NAC was removed, and conversion to a SSM was performed. After completion of superficial skin flap dissection, dissection of the peripheral portion of the breast parenchyma was performed. Posterior dissection was then performed by detaching the breast tissue from the pectoralis major muscle fascia with the perforator vessels clearly identified and secured. After completion of dissection, the entire breast specimen was removed intact through the incision.

#### Immediate implant/tissue expander reconstruction

In this study, suitable participants could opt for immediate reconstruction with an implant or tissue expander.

Following the removal of the specimen and adequate hemostasis, copious irrigation of the mastectomy pocket was performed. Subsequently, the lateral border of the pectoralis major muscle was elevated to allow submuscular pocket dissection. The working space was then developed under direct vision by electrocautery with the assistance of a handle light retractor. The single port was then re-inserted with CO_2_ insufflation for robotic submuscular pocket dissection using the da Vinci surgical platform. Dissection was performed medially towards the sternal border, taking care not to injure perforator vessels. Inferiorly, the dissection was performed beyond the inframammary fold over the lateral aspect, below which the muscle was released to continue the dissection to the subcutaneous plane, thus allowing for a more natural placement of the implant. In the lateral border, the superficial fascia of the serratus anterior muscle was dissected posteriorly in a limited fashion so that it would just be enough to accommodate the lateral border of the implant. After the initial dissection of the submuscular space with the da Vinci surgical platform, the robotic instruments and the single port were removed. The operating table was repositioned in the sitting position. The adequacy of the submuscular pocket dissection was checked and completed with the assistance of a light-source retractor. After the creation of the submuscular pocket, an implant (Mentor Worldwide LLC, Santa Barbara, CA, USA) was placed, followed by drain placement in both the submuscular and subcutaneous planes. An acellular dermal matrix or mesh may be used at the surgeon’s discretion.

### Outcome measures

Data collection included clinicopathological characteristics of patients, type of mastectomy, method of breast reconstruction (if any), intraoperative blood loss, surgical safety outcomes, and other outcome measures.

### Surgical outcomes

Operative parameters:
  - Docking time – defined as the time taken to dock the robot before robotic mastectomy.
  - Console time – defined as time taken for robotic mastectomy.
  - The total operative time was defined as the time taken from the robot docking time to the end of console time.
  - Length of stay – defined as the length of hospitalization from admission till discharge.
30-days morbidity/complication:
  - Wound infection requiring intervention: defined as wound infection where conservative treatment fails and requires surgical debridement.
  - Flap and NAC necrosis – defined as ischemia of the mastectomy skin flap or NAC necrosis.
  - Postoperative hematoma/bleeding requiring intervention was defined as immediate postoperative bleeding or hematoma requiring emergent exploration and hemostasis.
  - Anesthesia-related complications – defined as complications related to anesthesia conduct.

### Oncologic outcomes

Margin positivity is defined as margins involved or uninvolved. Margin involvement was defined as tumor-on-ink for invasive carcinoma and a 2 mm margin or less for ductal carcinoma in situ.

### Sample size justification

As a pilot study, the current flat rules of thumb for the overall pilot trial sample size of a subsequent two-armed trial to determine the current sample size^20,21^ were used. Hence, a minimum pilot sample size of 20 cases was recommended. For the sample size justification, the confidence interval for the one-proportion approach with the continuity correction method is used, which can identify a realistic uncertainty level about the safety endpoint based on binomial theory. A sample size of 20 subjects will produce an 80% confidence interval with a width equal to 28% (resulting in an 80% confidence interval of 6–34%) when the sample proportion is 20%. Sample size calculation was performed using the PASS software [PASS 14 Power Analysis and Sample Size Software (2015). NCSS, LLC. Kaysville, Utah, USA, ncss.com/software/pass]. In addition, based on a learning curve analysis of robotic mastectomy^11^, a minimum of 12 cases is required to reduce the operative time, and a pilot sample size of 20 will allow for meaningful interpretation and analysis of learning curve data in this pilot study.

### Statistical and analytical plans

Patient characteristics and outcome variables are reported using descriptive statistics. Continuous variables will be presented as mean, SD, median, first-quartile and third-quartile, minimum, and maximum, while binary and categorical variables will be reported as frequency and percentage. Ninety-five percent confidence intervals were generated for the primary and secondary endpoints. Statistical analyses were performed using SAS software (version 9.4; SAS Institute Inc., Cary, NC, USA).

### Trial registration

This study was retrospectively registered on ClinicalTrials.gov.

### Ethical approval

The respective institutional board review panel approved this study on May 21, 2022.

## Results

### Baseline demographics and tumor characteristics

From December 22, 2022, to December 15, 2023, a total of 29 robotic mastectomies were performed in 17 patients. Baseline patient and tumor characteristics are shown in Table 1. The mean age of the patients was 54.2±9.4 years old. All the patients included in this study fulfilled the inclusion and exclusion criteria. There were 12 bilateral and five unilateral cases. Two patients underwent a bilateral risk-reducing mastectomy in view of BRCA gene mutation, while 10 patients underwent a contralateral risk-reducing mastectomy in the same sitting position as an ipsilateral therapeutic surgery for breast cancer. None of the patients had any major or poorly controlled comorbidities. All patients in the current study had no previous history of smoking. Breast cup sizes were mainly A-B, with no ptosis. R-NSM was performed in 23 cases, while the remaining six cases were R-SSM. In terms of axillary staging procedure, SLNB was performed in 17 cases, in which one case had negative intraoperative frozen sections, but final histopathology revealed metastatic carcinoma deposits in one of the SLNs, and the patient underwent completion axillary clearance through the same incision as the index operation. Immediate reconstruction using (implant/tissue expander) was performed in 14 patients.

**Table 1:** Baseline demographics and tumour characteristics of patients enrolled in PRoMiSing I study.

Demographics and baseline tumour characteristics	Number of cases
Mean age (years) (mean±SD)	54.2±9.4
Total number of patients	17
Laterality	
Unilateral	5
Bilateral	12
Total number of robotic mastectomy cases	29
Indications	
Breast cancer	15
Risk-reducing mastectomy	14
Comorbidities	
No	11
Yes	6
Breast cup size	
A	12
B	14
C	3
Presence of ptosis	
No	25
Yes	4
Types of robotic mastectomy	
Nipple-sparing	23
Skin-sparing	6
Axillary staging procedure	
Sentinel lymph node biopsy	17
Axillary dissection (frozen section negative, final histopathology positive)	1
No axillary staging procedure	12
Reconstruction	
Yes	14
Direct-to-implant (dual plane, pre-pec with full ADM wrap)	8
Tissue expander	6
No	15

ADM, acellular dermal matrix; pre-pec, prepectoral.

### Surgical and oncological outcomes

In terms of operative parameters, the mean total operative time was 95±10.2 min, with docking time and console time being 10.9±1.5 min and 45±23.3, respectively. The average blood loss was 5.7±1.9 ml, and the average length of stay was 1.05 days. The mean weight of the mastectomy specimens was 251±55.4 g (Table 2).

**Table 2:** Surgical outcomes, oncological outcomes, and postoperative histopathology.

Outcomes	
Surgical outcomes	
Operative time (min) (mean±SD)	
Docking time	10.9±1.5
Console time	45±23.3
Total operative time	95±10.2
Average blood loss (ml)	5.7±1.9
Average length of stay (days)	1.05
	Number of cases
Conversion to open/conventional mastectomy	0
30-day morbidity/complication	
Wound infection requiring intervention	0
Flap and nipple–areolar complex (NAC) necrosis	0
Postoperative hematoma/bleeding requiring intervention	0
Anesthesia related complications	0
Oncologic outcomes	Number of cases
Margin involvement	
No	15
Yes	0
Not applicable	14
Mean mastectomy specimen weight (g)	251±55.4
Final histopathology stage	Number of cases
Stage 0	6
Stage 1	6
Stage 2	3
Invasive tumor size (mm)	27.1±21.8
Breast cancer subtype	Number of cases
Luminal-like	9
Basal-like	0
CerbB2 positive	0
Not applicable (DCIS)	6
Follow-up (months) (mean±SD)	4.5±3.3

There was no conversion to conventional mastectomy in any case. Furthermore, there were no 30-day morbidity or complications in terms of wound infection requiring intervention, flap and NAC necrosis, and postoperative hematoma/bleeding requiring intervention.

The oncologic outcome assessment in terms of margin involvement was 0%. Most patients had stages 0–1 disease with a mean invasive tumor size of 27.1±21.8 mm. The mean follow-up duration for this study was 4.5±3.3 months.

### Learning curve analysis

**Figure 1:**
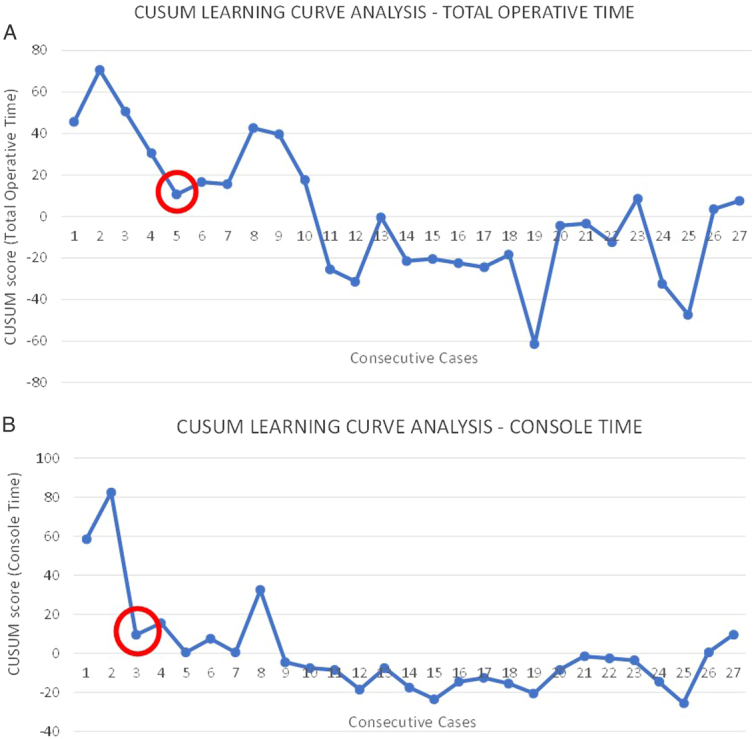
Learning curve analysis (CUSUM) of a single surgeon with formal fellowship training and experience in minimally invasive (endoscopic and robotic) breast surgery.

Learning curve analysis of 29 consecutive cases of R-NSM/R-SSM cases revealed a significant reduction in total operative time and console time in the fifth and third cases, respectively (Fig. 1).

## Discussion

The PRoMiSing I study is valuable as the first safety and feasibility prospective trial of robotic mastectomy in Singapore and Southeast Asia, and the results demonstrated the safety of this technique and its suitability in the study population. In terms of demographics and tumor characteristics, all patients included in this study had early breast cancer with small-to-moderately sized breasts. There were almost equal numbers of breast cancer (15 cases) and risk-reducing mastectomy (14 cases).

In terms of operative outcomes, there was 0% conversion to conventional mastectomy. In a single case where intraoperative frozen section analysis for SLN was negative, but final histopathology revealed 7 mm macrometastases, the patient successfully underwent completion axillary clearance through the same initial incision. Minimal blood loss (mean=5.7±1.9 ml) was observed in all cases of robotic mastectomy in this study. As reported in previous studies^5,15,18,22^, pneumomastia created with CO_2_ insufflation at 8 mmHg most likely contributes to minimal blood loss. The authors routinely reduced the pneumomastia to 6 mmHg to ensure that any bleeding was secured to prevent postoperative bleeding. There were no issues with subcutaneous emphysema, which could be attributed to accurate plane and boundary recognition, as well as dissection with the precision and ergonomics provided by a robotic surgical system. In terms of the NAC necrosis, 0% (0/23) was noted in all cases of NSM. Possible reasons have been reported and attributed to better preservation of the blood supply of the NAC with areolar-sparing incisions used in robotic mastectomy, as per previous studies^12,17,23^.

**Figure 2:**
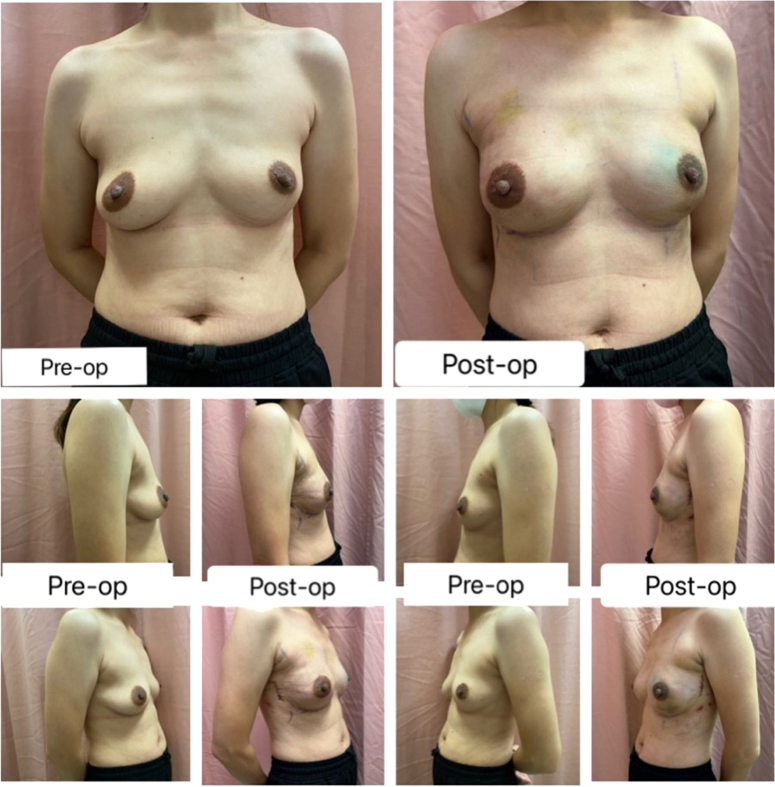
Esthetic outcomes of robotic mastectomy.

Implant or tissue expander reconstruction was the only reconstruction method used in this study, as the authors believed that this method maximized the advantage of robotic mastectomy, where a small incision could also accommodate implant/tissue expander placement with excellent esthetic outcomes (Fig. 2). Implant placement was either in the prepectoral or subpectoral plane with or without acellular dermal matrix placement. Indocyanine green was routinely used to assess mastectomy skin flap and NAC perfusion in this study to guide the decision to use tissue expanders in the event of unsatisfactory mastectomy skin flap perfusion. In patients who did not opt for reconstruction, the preference for robotic mastectomy was made based on a shared decision-making process with a preference for flat esthetic closure as well as small hidden incisions. In addition, suitable patients for this option would be ladies with small, nonptotic breasts.

Contrary to previously reported longer operation time as one of the limitations of R-NSM^8,10–12,16,24,25^, the operative timing in this study was noted to be acceptable as the mean docking time, console time, and total operative time was noted to be 10.9±1.5, 45±23.3, and 95±10.2 min, respectively. Furthermore, a learning curve analysis using the CUSUM method (Fig. 1) demonstrated that the learning curve of R-NSM/R-SSM in this study was two and five cases for console time and total operative time, respectively. The respectable operative time and short learning curve could be attributed to the fact that the main surgeon was trained and proficient in endoscopic breast surgery and assisted in at least 20 cases of R-NSM/R-SSM before embarking on robotic mastectomy. This further underlines the importance of adequate training and supervision to ensure excellent operative outcomes and safe robotic mastectomy.

In terms of limitations, the authors acknowledged that this was a small series performed by a single surgeon. In addition, the short follow-up duration in the current study was inadequate for assessing long-term outcomes, including oncological safety. However, as the main objective was to prove the safety and feasibility of robotic mastectomy in the study population and to serve as a pilot study for further studies, the results demonstrated the potential of robotic mastectomy as a safe alternative to conventional mastectomy. In addition, the learning curve analysis proved to be a valuable finding, as it further consolidated the finding that a surgeon trained in endoscopic breast surgery would have a shorter learning curve when embarking on robotic mastectomy.

## Conclusion

The PRoMiSing I study demonstrated the safety and feasibility of robotic mastectomy in Singapore and paved the way for robotic mastectomy in Southeast Asia. Further long-term prospective phase II cohort studies are in progress to ascertain the long-term oncological outcomes of robotic mastectomy.

## Ethical approval

Approval has been obtained from SingHealth Centralised Institutional Review Board (CIRB) in 2021 with the reference number 2021/2680.

## Consent

Written informed consent was obtained from the patient for publication of this study and accompanying images. A copy of the written consent is available for review by the Editor-in-Chief of this journal on request.

## Source of funding

This study was supported by the following grants: Changi General Hospital Joint Research Innovation Grant (JRIG) (Ref No.: RIG202110-005PR) and Changi General Hospital Ad Hoc Research Grant (FY2020 Breast Cancer Support Grant) (Ref No.: CHF2020.06-A).

## Author contribution

C.W.M.: study concept and design. C.W.M., Y.L.M.S., Z.C.L., and C.M.J.S.: data collection. C.W.M. and Y.L.M.S.: data analysis. C.W.M. and Y.L.M.S.: data interpretation. C.W.M., J.X.J.H., and S.M.T.: manuscript drafting. C.W.M., Z.C.L., and S.M.T.: manuscript writing.

## Conflicts of interest disclosure

The authors declare no conflicts of interest.

## Research registration unique identifying number (UIN)

ClinicalTrials.gov Identifier: NCT06335550.

## Guarantor

Chi Wei Mok and Yert Li Melissa Seet.

## Data statement

The data that support the findings of this study are not openly available due to sensitivity reasons and are available from the corresponding author upon reasonable request. Data are located in controlled access data storage at the authors’ institution.

## Provenance and peer review

Not commissioned, externally peer-reviewed.

## Presentation

None.
